# Supplementary material for: Association between Dietary Patterns and Handgrip Strength: Analysis of the Korean National Health and Nutrition Examination Survey Data Between 2014 and 2017
Source: Nutrients. 2020 Oct 5;12(10):3048. doi: 10.3390/nu12103048 (PMC7601920; doi:10.3390/nu12103048)
Supplement: Supplementary file 1 [file nutrients-12-03048-s001.pdf]

Table S1. General differences in 10–18-year-old children and adolescents according to gender and age.

	Gender			Age		
	All	Boys	Girls	10~12yr	13~15yr	16~18yr
N	2327	1223	1104	865	807	655
Age	14.2 ± 0.1	14.2 ± 0.1	14.2 ± 0.1	11.0 ± 0.0	14.1 ± 0.0	16.9 ± 0.0***
BMI	20.9 ± 0.1	21.1 ± 0.1**	20.6 ± 0.1	19.2 ± 0.1	21.1 ± 0.1	22.1 ± 0.2***
95th %tile ≤ BMI	329 (14.1%)	200 (16.3%)*	129 (12.6%)	77 (9.1%)	119 (13.7%)	133 (19.6%)***
HGS	26.6 ± 0.3	30.9 ± 0.4***	21.9 ± 0.2	18.2 ± 0.2	28.3 ± 0.3	32.0 ± 0.4***
SBP (mmHg)	108.4 ± 0.3	109.5 ± 0.34***	106.5 ± 0.4	106.3 ± 0.4	108.5 ± 0.4	109.9 ± 0.4***
DBP (mmHg)	66.4 ± 0.2	66.8 ± 0.3***	65.9 ± 0.3	62.8 ± 0.3	66.0 ± 0.3	69.6 ± 0.4***

Values are presented as mean ± *SE*; BMI: body mass index; HGS: handgrip strength; BP: blood pressure; SBP: systolic blood pressure; DBP: diastolic blood pressure. * *p*-values for differences using the analysis of variance for means; * $p < 0.05$, ** $p < 0.01$, *** $p < 0.001$.
